# _h_CeO_2_@ Cu_5.4_O nanoparticle alleviates inflammatory responses by regulating the CTSB–NLRP3 signaling pathway

**DOI:** 10.3389/fimmu.2024.1344098

**Published:** 2024-04-22

**Authors:** Ying Li, Xiaomin Xia, Zhaojun Niu, Ke Wang, Jie Liu, Xue Li

**Affiliations:** ^1^ Department of Stomatology, The Affiliated Hospital of Qingdao University, Qingdao University, Qingdao, China; ^2^ School of Stomatology, Qingdao University, Qingdao, China

**Keywords:** cerium oxide nanoparticles, copper-based nanoparticles, CTSB, NLRP3, anti-inflammation

## Abstract

Inflammatory responses, especially chronic inflammation, are closely associated with many systemic diseases. There are many ways to treat and alleviate inflammation, but how to solve this problem at the molecular level has always been a hot topic in research. The use of nanoparticles (NPs) as anti-inflammatory agents is a potential treatment method. We synthesized new hollow cerium oxide nanomaterials (_h_CeO_2_ NPs) doped with different concentrations of Cu_5.4_O NPs [the molar ratio of Cu/(Ce + Cu) was 50%, 67%, and 83%, respectively], characterized their surface morphology and physicochemical properties, and screened the safe concentration of _h_CeO_2_@Cu_5.4_O using the CCK8 method. Macrophages were cultured, and *P.g*-lipopolysaccharide-stimulated was used as a model of inflammation and co-cultured with _h_CeO_2_@Cu_5.4_O NPs. We then observe the effect of the transcription levels of CTSB, NLRP3, caspase-1, ASC, IL-18, and IL-1β by PCR and detect its effect on the expression level of CTSB protein by Western blot. The levels of IL-18 and IL-1β in the cell supernatant were measured by enzyme-linked immunosorbent assay. Our results indicated that _h_CeO_2_@Cu_5.4_O NPs could reduce the production of reactive oxygen species and inhibit CTSB and NLRP3 to alleviate the damage caused by the inflammatory response to cells. More importantly, _h_CeO_2_@Cu_5.4_O NPs showed stronger anti-inflammatory effects as Cu_5.4_O NP doping increased. Therefore, the development of the novel nanomaterial _h_CeO_2_@Cu_5.4_O NPs provides a possible new approach for the treatment of inflammatory diseases.

## 1 Introduction

Inflammation is the immune response that occurs when biological tissues are stimulated, which can be caused by infection or tissue damage. Inflammation can clear pathogens and promote tissue healing (1). However, the inflammatory response is sometimes the main cause of tissue damage—for example, if the stimulating factor persists and the inflammation does not subside, the infiltration of various inflammatory cells and the accumulation of inflammatory factors could alter the structure and function of normal tissues (2). Many studies have shown that inflammation is associated with many systemic diseases such as atherosclerosis (3), cancer (4), and diabetes (5). The complexity and unpredictability of inflammation make the treatment of inflammatory diseases a major challenge.

In recent years, nanotechnology has been developing rapidly, and nanoparticles are widely used in various fields, including in the monitoring and treatment of inflammatory diseases. There have been many reports in the literature that nanoparticles have anti-inflammatory properties and that they have better cell penetration than conventional drugs, resulting in better efficacy and durability in therapy (6–9). Therefore, nanomaterial-based drugs or drug carriers are proved to be a potential candidate for modulating inflammation (10–12).

There are increasing reports on the ability of nano-enzymes to treat different inflammatory diseases. It is worth mentioning that due to their unique valence state structure, cerium oxide nanoparticles (CeO_2_ NPs) have been found to possess various enzyme mimetic activities, thereby scavenging reactive oxygen species (ROS), including superoxide dismutase (SOD), catalase (CAT), oxidase-like activities, etc. (13) Cerium is a class of rare earth elements in the oxides of which trivalent (Ce^3+^) and tetravalent (Ce^4+^) states coexist. Due to this property, many studies have been conducted to evaluate the efficacy of CeO_2_ NPs in various inflammatory disease—for example, CeO_2_ NPs alleviated hypoxia-induced oxidative stress and inflammation in the lungs of mice (14). In another study, CeO_2_ NPs ameliorated inflammation in a model of periodontitis by reducing ROS production and inhibiting MAPK- NF-κB signaling (15).

Cu_5.4_O NPs is a new type of nano-enzymes synthesized in recent years. The ratio of Cu NPs to Cu_2_O NPs is 3.4 by controlling the temperature and the ratio of materials required for the synthesis, so it is named as Cu_5.4_O NPs. It has attracted attention because of its highly efficient catalytic performance and ultra-small particle size (16, 17). Copper, one of the essential trace elements for human growth and development, plays a key role in many cellular physiological processes (18). Cu NPs have excellent catalytic properties and can scavenge H_2_O_2_ and O_2_^•-^ but not •OH, whereas Cu_2_O NPs can react with both H_2_O_2_ and •OH. Therefore, it is speculated that Cu_5.4_O may have spectroscopic mimetic enzyme catalytic properties and antioxidant activity, which has been confirmed in previous studies: Cu_5.4_O NPs have a high scavenging efficiency against H_2_O_2_ (80%), O_2_^•-^ (50%), and •OH (80%) at a lower concentration (150–200 ng/mL) (16). Therefore, both CeO_2_ NPs and Cu_5.4_O NPs can be used as potential anti-inflammatory agents.

Pyroptosis is a recently discovered new type of programmed cell death that has the dual effect of protecting cells from endogenous and exogenous dangers and causing pathological inflammation (19). The classical activation pathway of pyroptosis is triggered by NLRP3 inflammasome. When it is stimulated by pathogen-associated molecular patterns or danger-associated molecular patterns, it could activate caspase-1, which could cause the release of proinflammatory factors such as IL-18 and IL-1β. Much of the literature suggested that the NLRP3 inflammasome is associated with various inflammatory diseases—for example, it is well known that LDL is a key factor in the formation of atherosclerosis, and it has been shown that phagocytosis of LDL induces the secretion of IL-1β through the activation of the NLRP3 inflammasome, which plays an important role in the development of atherosclerosis (20). Cathepsin B (CTSB) is a class of cysteine protein hydrolases found in lysosomes that primarily function as protein degraders and are involved in apoptosis (21). Recently, it has been found that CTSB is also involved in the pathological processes of many inflammatory diseases—for example, CTSB was significantly upregulated in coxsackievirus B3-induced myocarditis tissues, and CTSB knockout mice exhibited less inflammatory cell infiltration (22). It has also been found that CTSB expression was higher in the intestinal macrophages of mice with inflammatory bowel disease than in normal controls, and when CSTB was inhibited, only mild inflammation was shown in tissue sections (23).

**Scheme 1:**
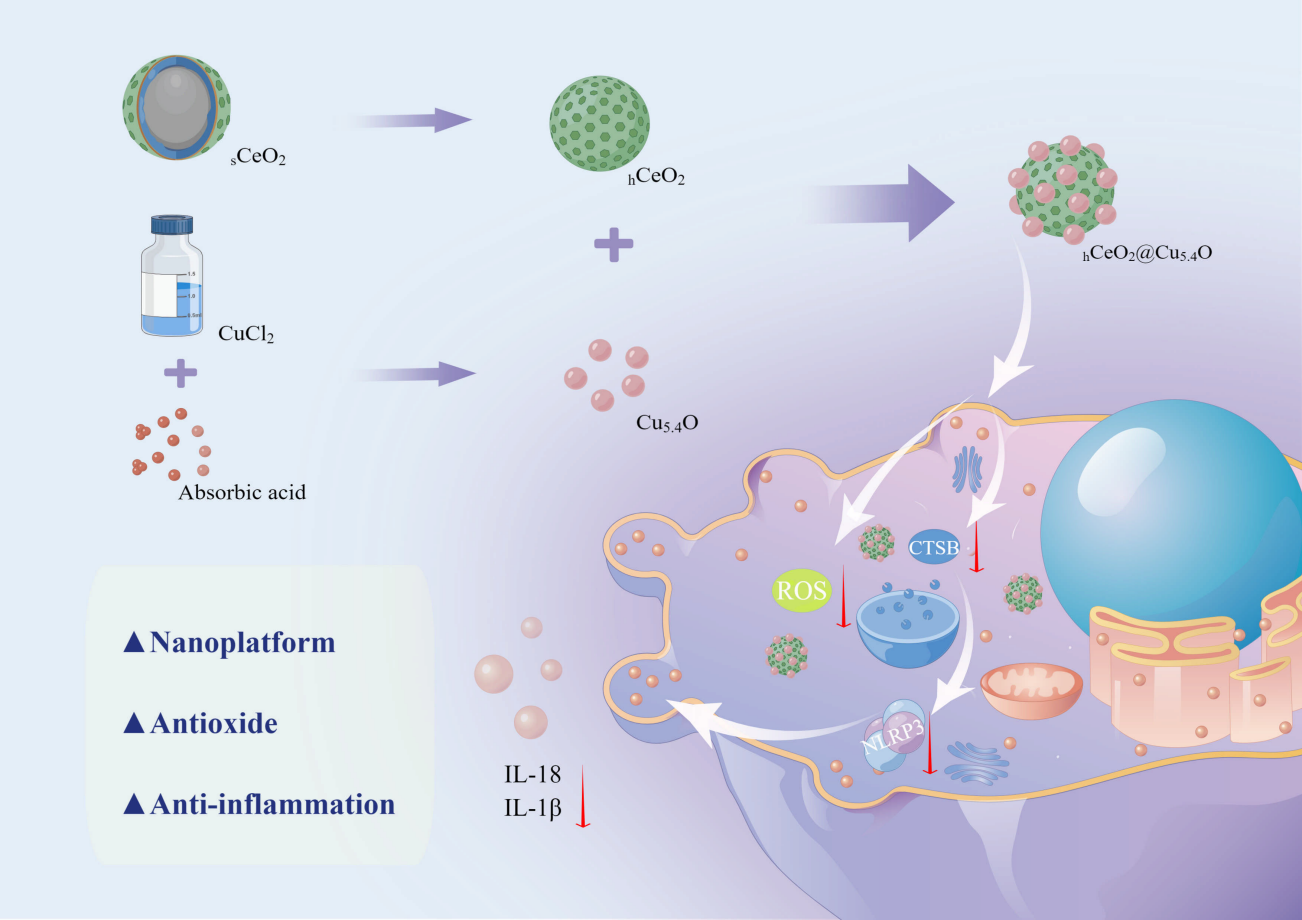
Schematic diagram of the synthesis process of _h_CeO_2_@Cu_5.4_O NPs and the mechanism of its anti-inflammatory effect. (By Figdraw).

In summary, CeO_2_ NPs and Cu_5.4_O NPs as nano-enzymes all have excellent antioxidant properties; they eliminate ROS in the inflammatory region and can be used as potential anti-inflammatory agents. In the study of the mechanism of inflammatory diseases, both CTSB and NLRP3 inflammasome have a promoting effect on inflammation. Therefore, we constructed a new nano-system, hollow cerium dioxide loaded with copper oxide (_h_CeO_2_@Cu_5.4_O NPs), and investigated whether its anti-inflammatory effects on macrophages could be mediated through the CTSB–NLRP3 signaling pathway (**Scheme 1**). The specific experiments include the following: (1) development of novel _h_CeO_2_ doped with different Cu/(Ce + Cu) molar ratios of 50%, 67%, and 83% and characterization of their physical and chemical properties using transmission electron microscopy (TEM), X-ray diffraction (XRD), and X-ray photoelectron spectroscopy (XPS). Determination of enzyme mimetic activity using antioxidant kit; (2) evaluation of the biosafety of _h_CeO_2_@Cu_5.4_O NPs by CCK8, live and dead cell staining, and hemolysis assay; and (3) evaluation of the inhibitory effect of _h_CeO_2_@Cu_5.4_O NPs on the secretion of inflammatory factors and its effect on the CTSB–NLRP3 pathway by RT-qPCR and Western blot methods. The experimental results showed that _h_CeO_2_@Cu_5.4_O NPs have good biocompatibility and broad-spectrum enzyme mimetic activity and are able to inhibit the CTSB–NLRP3 signal pathway in multiple ways. Therefore, this study could provide a feasible approach for the treatment of inflammatory diseases.

## 2 Materials and methods

### 2.1 Materials and reagents

Polyvinylpyrrolidone (PVP) was purchased from Ourchem (Shanghai, China). Tetraethoxysilane (TEOS) and cerium (III) nitrate hexahydrate [Ce(NO_3_)_3_·6H_2_O] were obtained from Macklin (Shanghai, China). Hexamethylenetetramine (HMTA), sodium hydroxide (NaOH), and copper (II) chloride dihydrate (CuCl_2_·2H_2_O) were purchased from Hushi (Shanghai, China). L-Ascorbic acid was purchased from Amethyst (Beijing, China). Cell Counting Kit-8 and CA-074Me were obtained from Dalian Meilun. AM/PI Double Staining Kit was purchased from Beyotime Biotechnology (Shanghai, China). ABclonal (Wuhan, China) supplied ABScript III RT Master Mix and SYBR Green Fast qPCR Mix. CathepsinB Rabbit mAb was purchased from Cell Signaling Technology (Boston, MA, USA). Beta-actin polyclonal antibody and goat anti-rabbit IgG were purchased from Elabscience Biotechnology Co., Ltd. (Wuhan, China). Reagent-grade water was obtained from ultra-pure water system (Ulupure, Chengdu, China) in all experiments. All other reagents were of analytical grade without further purification.

#### 2.1.1 Preparation of hollow CeO2 (_h_CeO2) NPs

The synthesis of hollow CeO_2_ (_h_CeO_2_) NPs was referred to a previous literature (24). In brief, 30 mL of absolute ethanol, 5 mL of 4 mol/L ammonia solution, and 4 mL of deionized water were put into an oil bath and mixed. When the above-mentioned solution was heated to 60°C, the mixture of 5 mL TEOS and 20 mL of absolute ethanol was slowly dripped into the mixture. The mixture was stirred at 60°C for 4 h. After cooling to room temperature, the mixture was washed three times with ethanol and dried under vacuum at 60°C to obtain silica (SiO_2_) NPs.

Then, 0.1 g silica and 1 g PVP were added to 40 mL of deionized water. When the oil bath was heated to 75°C, 5 mL of 0.5 mmol cerium nitrate and 5 mL of 0.5 mmol HMTA were added in turn. The mixture was stirred at 95°C for 2 h, washed and centrifuged three times after cooling, and dried to obtain SiO_2_@CeO_2_ core–shell (_s_CeO_2_) NP precursors.

The _s_CeO_2_ NP precursors were heated to 600°C at 5°C/min for 2 h, and then heating was naturally dropped to room temperature to obtain _s_CeO_2_ NPs.

Next, 0.1 g _s_CeO_2_NPs was dispersed in 40 mL of 2 mol/L sodium hydroxide and stirred for 24 h, centrifuged, washed three times with ethanol, and dried to obtain _h_CeO_2_ NPs.

#### 2.1.2 Preparation of Cu_5.4_O NP solution

The synthesis of Cu_5.4_O NPs was modified a little based on the previous literature (16). In detail, 10 mM CuCl_2_·2H_2_O was dissolved in 50 mL deionized water and stirred at 80°C in an oil bath for 10 min, and then 50 mL of 100 mM L-Ascorbic acid was added slowly to the above-mentioned solution. When the temperature of the solution is reduced to normal temperature, adjust the pH of the solution to 8 to 9 with 1 M NaOH solution and then stir for 12 h at 80°C. The large aggregates are then removed by centrifugation to obtain Cu_5.4_O NPs.

#### 2.1.3 Preparation of _h_CeO_2_@Cu_5.4_O NPs

Different masses of _h_CeO_2_ NPs were weighed and added to Cu_5.4_O NPs solution so that the molar ratio of Ce/Cu is 0.2:1, 0.5:1, and 1:1, respectively. They were referred to as _h_CeO_2_@83%Cu_5.4_O, _h_CeO_2_@67%Cu_5.4_O, and _h_CeO_2_@50%Cu_5.4_O. After stirring for 24 h, the precipitate obtained after centrifugation and drying is _h_CeO_2_@Cu_5.4_O NPs. Nanoparticles were obtained after washing three times using ethanol to remove impurities and then drying.

### 2.2 Dispersion and sterilization of nanoparticles

A total of 1 mg nanoparticles was weighed and exposed to UV light for 30 min, then dispersed in 10 mL of complete medium containing 10% FBS, stirred for 1 h at room temperature, and then diluted and stirred for another 24 h for subsequent experiments. The content of endotoxin in all dispersions was less than 0.5 EU/mL (_h_CeO_2_@83%Cu_5.4_O NPs: 0.453 EU/mL, _h_CeO_2_@67%Cu_5.4_O NPs: 0.444 EU/mL, and _h_CeO_2_@50%Cu_5.4_O NPs: 0.463 EU/mL) using the Chromogenic LAL Endotoxin Assay Kit (Beyotime, Shanghai). Dynamic light scattering (DLS) was carried out in suspensions using the zeta potentiometer (Zetasizer Nano ZS, England). The hydrated particle size and zeta potential of the nanoparticles were then calculated.

### 2.3 Characterization

The surface morphology of the nanoparticles was observed using transmission electron microscopy (HT7700, Japan), and particle size analysis of electron microscopy images of Cu_5.4_O NPs was performed using ImageJ. Elemental analysis of nanomaterials was carried out by X-ray diffraction (Xtalab Synergy, Netherlands) and comparison with standard mapping.

Analysis of the surface chemical composition and elemental valence of nanomaterials were determined by X-ray photoelectron spectroscopy (Smart Lab 3KW, Japan).

### 2.4 SOD, CAT, and T-AOC enzyme mimic activity

To evaluate the antioxidant properties of _h_CeO_2_@Cu_5.4_O NPs, we measured its superoxide dismutase (SOD), catalase (CAT), and total antioxidant (T-AOC) capacity using an enzyme calibrator (Elx800, Bio Tek, United States). The SOD, CAT, and T-AOC enzyme mimic activities of _h_CeO_2_@Cu_5.4_O NPs were measured by using SOD assay kit (Solebo, China), CAT assay kit (Solebo, China), and T-AOC assay kit (Solebo, China), respectively.

SOD is an enzyme found widely in plants, animals, and cells and catalyzes the disproportionation of superoxide anions to produce H_2_O_2_ and O_2_. The superoxide anion produced during metabolism reduces azotetrazolium to methyl salts, and SOD scavenges the superoxide anion, thereby reducing the formation of methyl salts and thus affecting its absorbance at 560 nm. The reagents were mixed thoroughly according to the instructions and divided into test group, control group, and two blank groups. A total of 18 μL 10 mg/L _h_CeO_2_@Cu_5.4_O NPs was added to the test group and the control group, and after 30 min of immersion in 37°C water bath, the absorbance value was measured at 560 nm, which was recorded as A test, A control, A blank 1, and A blank 2, respectively. The inhibition rate and SOD activity were calculated from the above-mentioned values.

CAT is an enzyme that mainly scavenges H_2_O_2_, and the absorbance value at 240 nm of the reaction solution changes when H_2_O_2_ is decomposed, thus calculating the CAT activity. Specifically, the CAT detection working solution was bathed at 37°C for 10 min, and 1 mL of the above-mentioned liquid was added to 35 μL of 10 mg/L _h_CeO_2_@Cu_5.4_O NPs. After mixing, the absorbance value at 240 nm was measured immediately, and then the absorbance value after 1 min was measured, and the CAT activity was calculated according to the absorbance value.

The total antioxidant level of the nanoparticles was determined. The total antioxidant capacity of the samples was calculated by measuring the amount of Fe^3+^-TPTZ reduced to Fe^2+-^TPTZ in an acidic environment. Furthermore, 6 μL 10 mg/L _h_CeO_2_@Cu_5.4_O NPs was mixed with 180 μL working solution and 18 μL distilled water, and the 593-nm absorbance value was measured after reacting at room temperature for 10 min. The 593-nm absorbance value was measured after the working solution without nanoparticles was mixed with distilled water for 10 min and substituted into the formula to determine the total antioxidant capacity.

### 2.5 Determination of intracellular ROS

The ROS assay kit (Beyotime, Shanghai) was used to measure the ROS in cells treated with lipopolysaccharide (LPS) and nanoparticles. In short, RAW 264.7 cells were seeded into six-well plates at 3 × 10^4^ cells per well and divided into six groups: the first group was the blank control group. The second group was treated with *P.g*-LPS (1 μg/mL) for 3 h to establish an *in vitro* inflammation model. The third group was pretreated with cathepsin B inhibitor (CA-074Me) for 2 h and then treated with LPS for 3 h as a positive control group. Four to six groups were treated with *P.g*-LPS followed by the addition of safe concentrations (10 mg/L) of the three groups of drugs for 24 h. The medium containing nanoparticles was then replaced with serum-free medium supplemented with 10 uM DCFH-DA and incubated at 37°C for 30 min. The cells were washed three times with serum-free medium and placed under an inverted fluorescence microscope for observation.

### 2.6 Cell culture

Mouse leukemia cells of monocyte macrophage (RAW264.7) were purchased from American Type Culture Collection (ATCC, Manassas, VA, USA) and cultured in Dulbecco’s modified Eagle’s medium (DMEM) with 10% fetal bovine serum (FBS). The L929 cell line was purchased from ScienCell (SanDiego, CA, USA) and cultured in DMEM with 10% FBS, 10,000 U/mL penicillin, and 10 mg/mL streptomycin. All cell lines were cultured at 37°C in an incubator with 5% CO_2_. The cells were passaged when the cell density reached 80%–90%.

### 2.7 Cytotoxicity assay

#### 2.7.1 Cytocompatibility test

The cytotoxicity of the nanoparticles was evaluated by using Cell Counting Kit-8 (Shanghai St Er), and the safe concentration was screened for subsequent experiments. L929 cells were seeded into 96-well plates at 5 × 10^3^ cells per well. After cell adhesion, different concentrations (10, 20, 30, 40, and 50 mg/L) of nanoparticles were added to the culture for 24, 48, and 72 h. The medium-containing nanoparticles were removed and washed three times with PBS. Then, 10% CCK8 reagent was added, and the cells were incubated in an incubator at 37°C for 0.5–3 h in the dark. The absorbance at 450 nm was then determined using a microplate reader (Bio-Tek, Winooski, VT, USA). Five parallel wells were set up for each group, and the experiment was repeated three times.

After treatment of the nanoparticles, the cells were washed three times with PBS and incubated with calcian-AM and PI for 30 min before being observed under a fluorescence inverted microscope.

#### 2.7.2 Hemolysis test

For the hemolysis assay, fresh blood was collected from mice and added with anticoagulant and saline to test the hemolytic potential of nanoparticles. Blood diluted with distilled water was used as a positive control, and blood diluted with saline was used as a negative control. The cells were incubated at 37°C for 4 h and centrifuged at 2,500 RPM for 10 min, the supernatant was removed, and the absorbance at 545 nm was recorded using a microplate reader. Hemolysis rates were calculated according to the following formula:


$$\text{Hemolysis rate }=\;\left(\text{O}{\text{D}}_{\text{exper}}-\text{O}{\text{D}}_{\text{negative}}\right)/\left(\text{O}{\text{D}}_{\text{positive}}-\text{ O}{\text{D}}_{\text{negative}}\right)$$

where OD_exper_, OD_negative_, and OD_positive_ represent the measured absorbance of the nanoparticle sample, negative control, and positive control, respectively.

#### 2.7.3 Cytocompatibility test with LPS and _h_CeO_2_@Cu_5.4_O co-treatment

To assess the toxicity of the combined treatment with LPS and nanoparticles (10 mg/L), CCK8 was used to evaluate the cytotoxicity. RAW cells were seeded into 96-well plates at a density of 5 × 10^3^ cells per well. After cell adhesion, LPS was added to treat the cells for 3 h, and then the cells were treated with the medium containing 10 mg/L nanoparticles for 24/48/72 h. After removal of the medium, the cells were washed three times with PBS, and the cells were added with 10% CCK8 reagent and incubated at 37°C in the dark for 0.5–3 h. The absorbance at 450 nm was measured using a microplate reader. To assess the effect of nanoparticles on absorbance at 450 nm, 100 uL of 10 mg/L _h_CeO_2_@83%Cu_5.4_O, _h_CeO_2_@67%Cu_5.4_O, and _h_CeO_2_@50%Cu_5.4_O was added to a 96-well plate. CCK8 reagent was added to a concentration of 10% and incubated at 37°C in the dark for 0.5–3 hours. The absorbance at 450 nm was measured using a microplate reader. Five parallel wells were set up for each group, and the experiment was repeated three times.

### 2.8 Uptake and intracellular localization of _h_CeO_2_@Cu_5.4_O NPs

After 24 h of treatment with _h_CeO_2_@Cu_5.4_O NPs, RAW264.7 cells were washed three times with PBS, and cell precipitates were collected. These were fixed with 2.5% glutaraldehyde at 4°C for 24 h, washed three times with PBS, and then fixed with 1% osmic acid for 1 to 2 h. Finally, the samples were dehydrated with gradient concentrations of ethanol and propanol and were treated overnight with an embedded agent. The sections were then double-stained with lead citrate–uranyl acetate, and the slices were imaged using Hitachi HT-7800.

### 2.9 Real-time PCR

Cells were grouped and treated as in Section 2.5. Then, the expressions of NLRP3 pathway-relative factors, TNF-α and TGF-β, were measured using quantitative real-time PCR. We also determined gene expression in cells treated with LPS and nanoparticles for 12 h as well as nanoparticles alone for 24 h.

The total RNA of RAW264.7 was extracted by using RNA-Easy (Vazyme). RNA was reverse-transcribed into cDNA using a reverse transcription kit (ABScript III RT Master Mix for qPCR with gDNA Remover, ABclomal). RT-qPCR was performed using Universal SYBR Green Mix (ABclomal), cDNA, and primers under the following conditions: 95°C for 5 s and 60°C for 30 s with 40 cycles. β-Actin was an internal control of genes. Data results were analyzed with 2^−ΔΔCt^. The primer sequences were as shown in **Table 1**.

**Table 1:** Primer sequences used in this study.

Gene	Forward sequence (5′ to 3′)	Reverse sequence (5′ to 3′)
β-Actin	CATCCGTAAAGACCTCTAGCCAAC	ATGGAGCCACCGATCCACA
IL-1β	TCCAGGATGAGGACATGAGCAC	GAACGTCACACACCAGCAGGTTA
IL-18	TGGCTGCCATGTCAGAAGACT	CCAGGTCTCCATTTTCTTCAGGT
CTSB	CTTCCCATGTCGGCAATCAG	GTGTAGTTGAGACCGGTGGA
NLRP3	CCTGACCCAAACCCACCAGT	TTCTTTCGGATGAGGCTGCTTA
ASC	AGAGACATGGGCTTACAGGAGC	CCACAAAGTGTCCTGTTCTGGC
Caspase-1	TGCCGTGGAGAGAAACAAGGA	TGGTGTTGAAGAGCAGAAAGCA
TNF-α	ACTCCAGGCGGTGCCTATGT	GTGAGGGTCTGGGCCATAGAA
TGF-β	CTTCAGCCTCCACAGAGAAGAACT	TGTGTCCAGGCTCCAAATATAG

### 2.10 Western blot assay

The cells were grouped and treated as in Section 2.5. After 24 h of treatment with nanoparticles, total proteins were extracted using RIPA buffer (Elabscience Biotechnology Co., Ltd.). The proteins were separated using 10% gel (Epizyme, shanghai) and then transferred to PVDF membranes (Solarbio, Beijing). After blocking with fast blocking western (Solarbio, Beijing) for 10 min at room temperature, PVDF membranes were incubated with primary antibodies (CST, 1:1,000) overnight at 4°C. β-Actin (Elabscience, 1:1,000) was used as an internal reference. The membranes were then incubated with the secondary antibody HRP goat anti-rabbit IgG (Elabscience, 1:5,000) for 1 h. Finally, each group of proteins was detected using electrochemiluminescent ECL reagents. The protein bands were quantified using Image software.

### 2.11 Immunofluorescence

RAW264.7 was seeded on the coverslips of a 24-well plate at a density of 5 × 10^4^ cells per well, treated as described above, washed three times with PBS, and then post-fixed with 4% paraformaldehyde (Elabscience, Wuhan) for 15 min, followed by permeabilization with 0.2% Triton X-100 for 15 min and blocking with 10% blocking serum (Solarbio, Beijing) for 60 min. Anti-CTSB primary antibody (CST, 1:500) was applied to the cells and left overnight at 4°C. The cells were then treated in the dark with secondary antibody FITC conjugated goat anti-rabbit IgG (Elabscience, 1:100) for 1 h. Finally, DAPI staining solution (Solarbio, Beijing) was added for 10 min at room temperature. Subsequently, the DAPI staining solution was removed, and the cells were washed with PBS, blocked, and observed using an inverted fluorescent microscope.

### 2.12 Enzyme-linked immunosorbent assay

After completion of the cell treatment, the cell supernatant fluid was collected. IL-18 and IL-1β content, respectively, in cell supernatant fluid were determined using an enzyme-linked immunosorbent assay (ELISA) kit (Reed Biotech, Wuhan) according to the manufacturer’s instructions.

### 2.13 Statistical analysis

All experiments were repeated three times, and data were analyzed using one-way ANOVA and Tukey’s multiple-comparison test in GraphPad software, with a single asterisk indicating significant differences between data (*p*< 0.05) and two and more asterisks indicating a strong difference between data (*p*< 0.01). The data in the graphs represent mean ± standard deviation.

## 3 Results and discussion

### 3.1 Characterization

**Figure 1:**
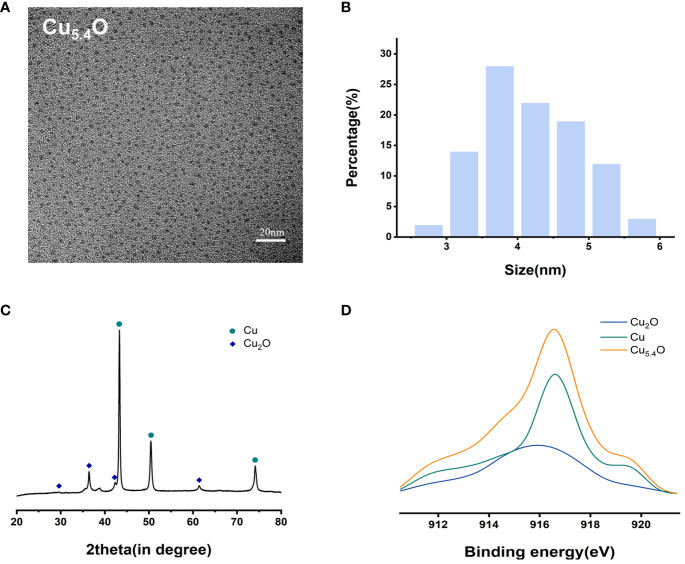
Characterization of Cu_5.4_O nanoparticles. **(A)** TEM images. **(B)** Particle size distribution. **(C)** XRD analysis. **(D)** XAES spectra.

From the transmission electron microscopy (TEM) images and particle size analysis (**Figures 1A, B**), it can be seen that the synthesized Cu_5.4_O NPs were homogeneously dispersed and morphologically uniform in solution, mostly between 3 and 5 nm in diameter. As shown in **Figures 1C, D**, the oxidation state of copper was studied by X-ray diffraction (XRD) pattern and X-ray photoelectron spectroscopy (XPS). The dominant peaks 2*θ* = 43.3°, 50.4°, and 74.2° correspond to (111), (200), and (220) in the copper lattice structure, and the other peaks 2*θ* = 29.6°, 36.4°, 42.3°, and 61.3° correspond to (110) (111), (200), and (220) in the copper oxide lattice structure, respectively. The XPS spectrum of the Cu_5.4_O NPs in **Figure 1D** also suggested that Cu_5.4_O NP was a mixture of Cu NPs and Cu_2_O NPs, and the ratio of Cu to Cu_2_O can be calculated to be approximately 3.4 based on the peak area and mass fraction of both. All of the above-mentioned data were consistent with previous reports in the literature (16, 24).

**Figure 2:**
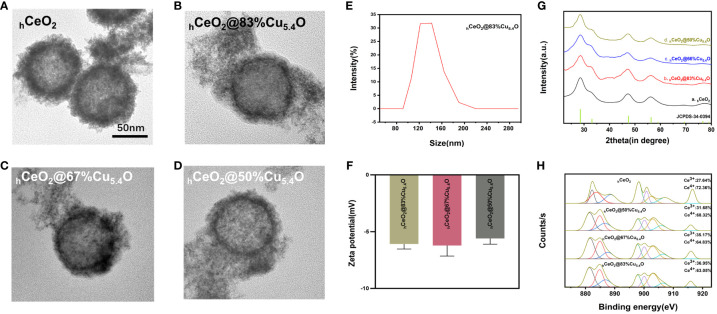
Characterization of _h_CeO_2_ and _h_CeO_2_@Cu_5.4_O nanoparticles (NPs). **(A)** TEM image of _h_CeO_2_ NPs. **(B)** TEM image of _h_CeO_2_@83%Cu_5.4_O NPs. **(C)** TEM image of _h_CeO_2_@67%Cu_5.4_O NPs. **(D)** TEM image of _h_CeO_2_@50%Cu_5.4_O NPs. **(E)** Particle size distribution of _h_CeO_2_@83%Cu_5.4_O NPs. **(F)** Zeta potential analysis of _h_CeO_2_@Cu_5.4_O NPs. **(G)** XRD analysis of _h_CeO_2_@Cu_5.4_O NPs. **(H)** XPS analysis of _h_CeO_2_@Cu_5.4_O NPs.

The TEM images of _h_CeO_2_ and _h_CeO_2_@Cu_5.4_O NPs are shown in **Figures 2A–D**. In **Figure 2A**, it can be seen that _h_CeO_2_ has an obvious hollow structure and a rough surface with a diameter of approximately 80–100 nm. **Figures 2B–D** were _h_CeO_2_@83%Cu_5.4_O NPs, _h_CeO_2_@67%Cu_5.4_O NPs, and _h_CeO_2_@50%Cu_5.4_O NPs, respectively. **Figures 2B–D** showed that _h_CeO_2_ doped with Cu_5.4_O NPs does not change the hollow form of _h_CeO_2_, and there were scattered Cu_5.4_O NPs around _h_CeO_2_. According to DLS data (**Figure 2E**), the average particle size of _h_CeO_2_@83%Cu_5.4_O NPs is approximately 140.3 nm, the zeta potential of _h_CeO_2_@83%Cu_5.4_O NPs, _h_CeO_2_@67%Cu_5.4_O NPs, and _h_CeO_2_@50%Cu_5.4_O NPs is -6.08, -6.21, and -5.59 mV, respectively (**Figure 2F**). The zeta potential is closely related to the stability of the dispersion. In general, the greater the absolute value of the zeta potential, the better the stability of the dispersion (25). As shown in **Figure 2G**, the XRD test of the synthesized _h_CeO_2_ NPs was consistent with the typical cerium spectra (JSPDS-34-0394), confirming its cubic fluorite structure. It is noteworthy that the XRD tests of three other samples doped with different proportions of Cu_5.4_O NPs were also consistent with _h_CeO_2_ NPs, which indicated that Cu_5.4_O NPs was in a highly dispersed or doped state into the _h_CeO_2_ NPs lattice or the yield of Cu_5.4_O NPs in the mixed product of both is relatively low. As shown in **Figure 2H**, the elemental valence states of _h_CeO_2_ NPs were analyzed, and the peaks at 885.0 and 903.5 eV in the XPS test belong to Ce^3+^, the peaks at 882.1, 888.1, 898.5, 900.9, 906.4, and 916.4 eV were in Ce^4+^, and the coexistence of the two indicates that it has peroxidase-mimicking activity and superoxide dismutase mimetic activity potential. In addition, after quantitative calculations, the percentage of Ce^3+^ in _h_CeO_2_ was 27.64%, and the percentages of Ce^3+^ in _h_CeO_2_@50%Cu_5.4_O NPs, _h_CeO_2_@67%Cu_5.4_O NPs, and _h_CeO_2_@83%Cu_5.4_O NPs were 31.68%, 35.17%, and 36.95%, respectively. The content of Ce^3+^ showed an increasing tendency with the increase of Cu_5.4_O NP incorporation.

ROS play an important role in the development of inflammation by inducing oxidative stress. It has been documented that ROS activates NF-κB signaling and NF-κB, as an upstream signaling molecule, stimulates histone TB release and NLRP3 inflammasome activation and promotes the aggregation of inflammatory cells and the expression of inflammatory factors (26, 27). Therefore, scavenging ROS is crucial for inflammatory diseases, and nanomaterials with enzyme-mimicking activity have excelled in the field of scavenging ROS in recent years. Therefore, various enzymatic activities and total antioxidant properties of the nanoparticles were further investigated.

CeO_2_ NPs have attracted much attention as a nano-enzyme, which has good biocompatibility and a variety of enzyme mimetic activities (28). The antioxidation property of CeO_2_ NPs is mainly related to the coexistence of two valence states (Ce^3+^/Ce^4+^) on cerium surface and mainly depends on the proportion of valence states (29). Superoxide dismutase (SOD), an enzyme capable of scavenging superoxide anion, is considered as a ROS detoxification enzyme because it can convert superoxide anion to H_2_O_2_ with low oxidation efficiency (30). The SOD enzymatic pseudo-activity of CeO_2_ NPs is achieved by the valence state conversion of Ce^3+^ (reduced state) and Ce^4+^ (oxidized state). The superoxide anion oxidizes Ce^3+^ to Ce^4+^, and the superoxide anion is reduced to H_2_O_2_. CAT enzyme mimetic activity is accomplished by the reaction of Ce^4+^ with H_2_O_2_ and its decomposition to H_2_O and O_2_, which protects cells from H_2_O_2_ damage while Ce^4+^ is converted to Ce^3+^ (31). This ability to switch between two oxidation states, Ce^3+^ and Ce^4+^, endows CeO_2_ NPs with regenerative properties.

There have been many studies utilizing Ce^3+^/Ce^4+^ interconversion on the surface of CeO_2_ NPs to scavenge ROS for the treatment of arthritis and inflammatory bowel disease (32–34). It has been suggested that the mechanism of CeO_2_ NP antioxidant is related to the presence of Ce^3+^, which makes the oxygen vacancies come out (35). It was reported that as the particle size of CeO_2_ NPs decreases, the Ce^3+^ on its surface gradually increases and its antioxidant effect is enhanced (35). Another research found that cerium dioxide nanocubes (0.17 μg/mL) containing a higher amount of Ce^3+^ (63%) were more effective in the ROS scavenging efficiency in HUVEC than that of CeO_2_ NPs (2.0 μg/mL) with a lower percentage of Ce ^3+^ (49%) than on the surface (36). Thus, the higher the Ce^3+^/Ce^4+^ on the CeO_2_ NP surface, the higher the concentration of defects and oxygen vacancies in the lattice with higher superoxide dismutase mimetic activity, and the better it is able to cope with oxidative stress and inflammatory diseases. As shown in **Figure 2H**, the doping of Cu_5.4_O NPs increased the Ce^3+^ content of _h_CeO_2_@Cu_5.4_O NPs. When the Cu/(Ce+Cu) molar ratio was 83%, the Ce^3+^ content of _h_CeO_2_@Cu_5.4_O NPs was 36.95%, which was an increase of 9.31% compared with that of _h_CeO_2_ NPs, which was more favorable for the scavenging of ROS, leading to better treatment of inflammatory diseases.

Cu, as a trace element in the human body, participates in many enzymatic reactions and brings a large surface area to volume ratio due to its small size, which leads to the appearance of surface effects (37). As for the antioxidant properties of Cu_5.4_O NPs, a study conducted XPS tests before and after its treatment with H_2_O_2_ and showed that the two peaks corresponding to Cu^+^ and Cu^0^, Cu 2p2/3 and Cu2p1/2 were not shifted, and a few new peaks appeared. Therefore, it is speculated that the ROS scavenging performance of Cu is mainly attributed to its inherent multi-enzyme mimetic properties (16). In one study, Cu_5.4_O NPs were found to protect cells from 250 μM H_2_O_2_ at a very low concentration (25 ng/mL), and the mRNA levels of antioxidant genes were all significantly increased in the kidneys of Cu_5.4_O NP-treated mice. The phosphorylation of NF-κB and IκB was significantly reduced. The expression of NF-κB signaling pathway downstream inflammatory factor expression was also decreased, suggesting that Cu_5.4_O NPs could protect renal tissues from oxidative stress by reducing the production of pro-inflammatory factors (16). It has also been shown that Cu_5.4_O NPs were combined with hydrogels to investigate their effects on wound healing, and the results indicated that Cu_5.4_O@Hep-PEG inhibited the expression of pro-inflammatory factors, scavenging of ROS, and promoting wound healing. In addition, Cu_5.4_O@Hep-PEG also promoted cellular cell proliferation as well as angiogenesis due to the presence of Cu_5.4_O NPs (38).

**Figure 3:**
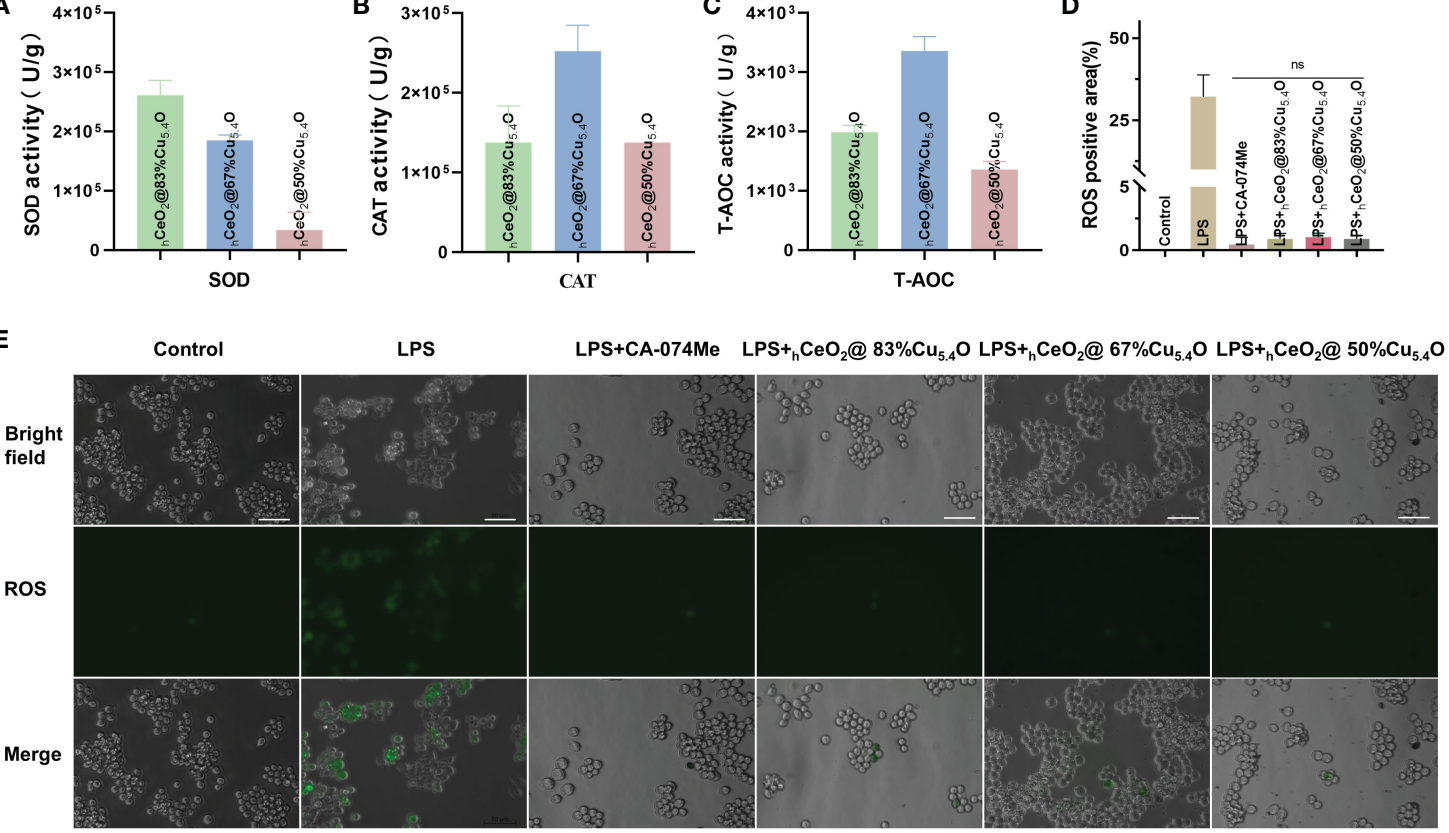
Ability of _h_CeO_h_@Cu5._4_O nanoparticles (NPs) to scavenge reactive oxygen species (ROS). **(A)** SOD enzymatic activities of _h_CeO_h_@Cu5._4_O NPs. **(B)** CAT enzymatic activities of _h_CeO_h_@Cu5._4_O NPs. **(C)** T-AOC enzymatic activities of _h_CeO_h_@Cu5._4_ONPs. **(D, E)** Intracellular ROS levels in RAW 264.7 cells treated with lipopolysaccharide (LPS) alone and the combined application of 10 mg/L _h_CeO_h_@Cu5._4_ONPs. Scale bar: 50 mm. (n=3; ns: no significance.)

In this study, as shown in **Figure 3A**, the SOD performance decreases with the reduction of Cu_5.4_O NP doping, so we speculated that the SOD activity of Cu_5.4_O NPs plays a major role in the composite particles, Alternatively, when the amount of doped Cu is less, the less Ce^4+^ is reduced to Ce^3+^. In **Figures 3B, C**, the three groups of different proportions of nanoparticles showed great CAT and total antioxidant properties, with the best CAT and total antioxidant properties at a molar ratio of (Cu/Ce+Cu) of 67%. Simple _h_CeO_2_ and Cu_5.4_O NPs showed good enzymatic activity, and Cu^+^ was easy to react with Ce^4+^ to generate Ce^3+^ and Cu^2+^ after Cu_5.4_O NP doping. From the discussion above, it can be seen that when the ratio of Ce^3+^/Ce^4+^ is large, CeO_2_ NPs showed better SOD mimics activity. Therefore, when the molar ratio of Cu/(Ce+Cu) was 83%, the nanoparticles exhibited the most excellent SOD-mimicking activity, which is more conducive to scavenging ROS, eliminating oxidative stress, and treating inflammatory diseases.

### 3.2 _h_CeO_2_@Cu_5.4_O NPs protect RAW264.7 cells from ROS damage

ROS are inevitable by-products of cellular metabolism, including superoxide anion (O_2_^•−^), hydroxyl radical (^•^OH), hydrogen peroxide (H_2_O_2_), etc., but endogenous ROS are extremely susceptible to interact with biomolecules and cause intracellular oxidative stress and DNA damage (39, 40). Oxidative stress, in general, can trigger inflammation, and excessive inflammation can, in turn, cause oxidative stress, inducing damage to cellular and tissue structure and function (41). In addition, it has been shown that ROS promotes the disruption of lysosomal membrane integrity and lysosomal membrane permeabilization (LMP) by initiating phospholipase A2 and activating lysosomal Ca^2+^ channels to allow the leakage of intra-lysosomal enzymes such as CTSB into the cytoplasm (42). Furthermore, ROS activates NF-κB signaling, and NF-κB, as an upstream signaling molecule, stimulates histone TB release and NLRP3 inflammasome activation and promotes the aggregation of inflammatory cells and the expression of inflammatory factors (26, 27). In summary, ROS plays an important role in the development of inflammation by inducing oxidative stress, increasing lysosomal membrane permeability to promote CTSB release, and activating NLRP3 inflammasomes, thereby promoting inflammation. Therefore, scavenging ROS is crucial for inflammatory diseases, and nanomaterials with enzyme-mimicking activity have excelled in the field of scavenging ROS in recent years.

In this study, we determined ROS scavenging due to the antioxidant properties of _h_CeO_2_@Cu_5.4_O. As shown in **Figures 3D, E**, the ROS levels were confirmed by using inverted fluorescence microscopy, and the results showed that the fluorescence intensity of cells treated with nanoparticles was reduced, and there was no significant statistical difference between the cells treated with the three groups of nanoparticles and the CA-074Me-treated group. We thus demonstrated that nanoparticles are able to scavenge ROS *in vitro*.

### 3.3 Biosafety assessment

Studies have shown that nanoparticles, especially metal-based nanoparticles, may cause a variety of adverse reactions in cells, such as oxidative stress and cell death (43). As a novel nanomaterial, biosafety evaluation is essential for biomedical applications. Previous studies have shown that hollow cerium dioxide has no significant cytotoxicity at concentrations ranging from 0 to 200 mg/L (24). However, studies on the toxicity of Cu_5.4_O NPs are limited, with articles stating no significant cytotoxicity at 600 ng/mL (38).

**Figure 4:**
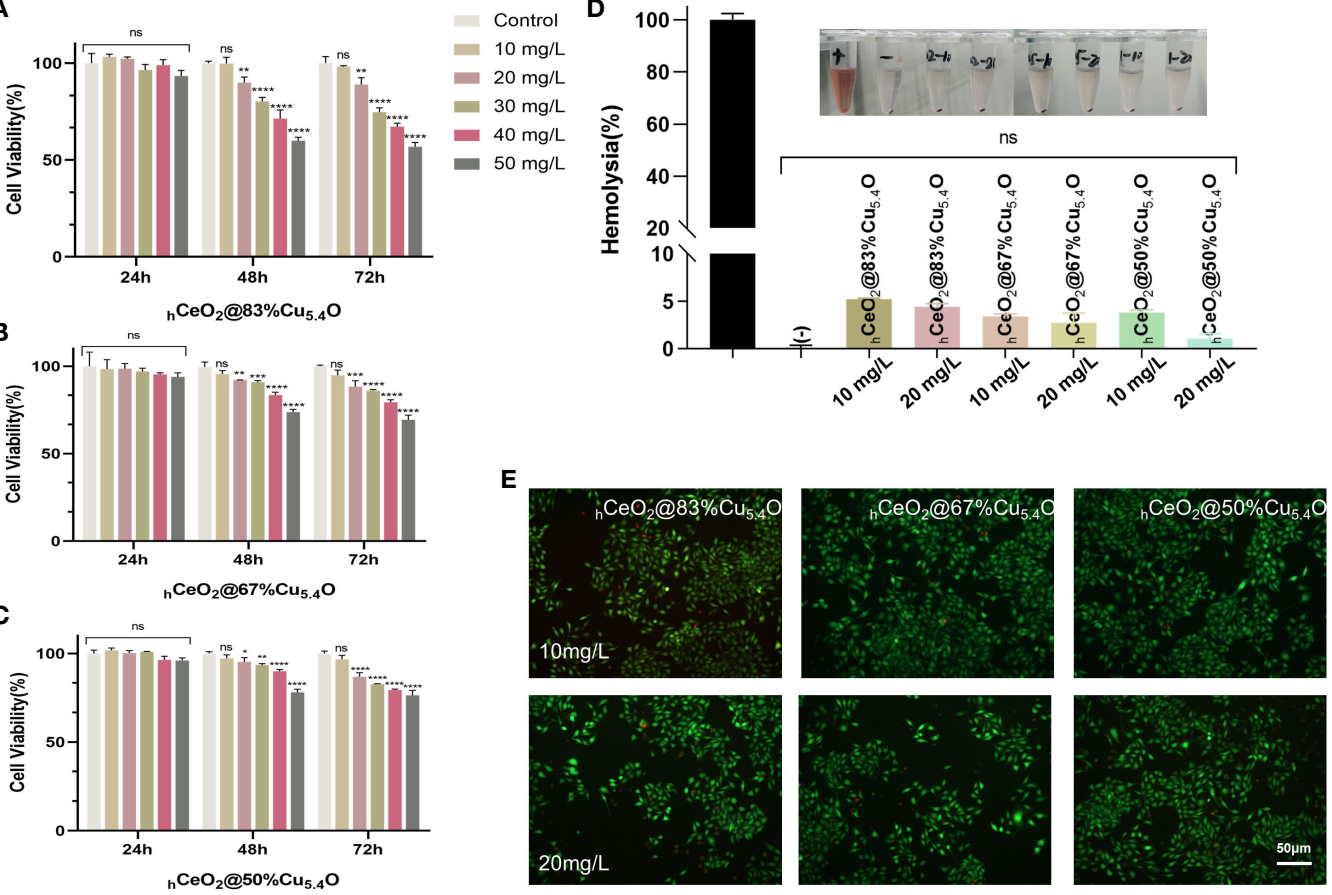
Biocompatibility of _h_CeO_2_@Cu_5.4_O nanoparticles (NPs). **(A–C)** The 24, 48-, and 72-h cytocompatibility of _h_CeO_2_@Cu_5.4_O NPs with the molar ratio of Cu/(Ce+Cu) was 83%, 67%, and 50%, respectively. **(D)** Blood compatibility of _h_CeO_2_@Cu_5.4_O NPs. (+) and (-) represent positive and negative controls, respectively. **(E)** Live/dead fluorescence staining images of fibroblasts treated with nanoparticles for 24 and 48 h. Data represent mean ± SD (*n* = 5; ns: no significance; * represents significant differences. *P< 0.05, **P < 0.005, ***P < 0.0005, ****P < 0.0001).

In this experiment, cytocompatibility and blood compatibility were used to analyze the biocompatibility of nanoparticles. In the CCK8 assay, L929 was used to evaluate the cytocompatibility of the nanoparticle. **Figures 4A–C** were the results of the CCK8 experiment with the molar ratio of (Cu/Ce+Cu) of 83%, 67%, and 50%, respectively. It can be seen that, with the decrease of Cu_5.4_O NPs, the activity of cells treated with nanoparticles increases gradually, which indicated that the toxicity of nanoparticles was mainly attributed to the doped Cu_5.4_O NPs. In addition, in each group, the cytotoxicity of 0–50 mg/L nanoparticles was tested. The results showed that all three groups of nanoparticles showed excellent cytocompatibility within 24 h, and no significant difference was observed. However, at 48 and 72 h, significant cytotoxicity was observed when the concentration was higher than 10 mg/L, and the cytotoxicity increased with the increase of the concentration. Similar results were obtained with live or dead staining (**Figure 4E**). With respect to blood compatibility, the hemolysis results (**Figure 4D**) showed good blood compatibility of the nanoparticles. In conclusion, our synthesized nanoparticles showed excellent biocompatibility at 10 mg/L, and a safe concentration of 10 mg/L was used for all three proportions of drugs in subsequent experiments.

**Figure 5:**
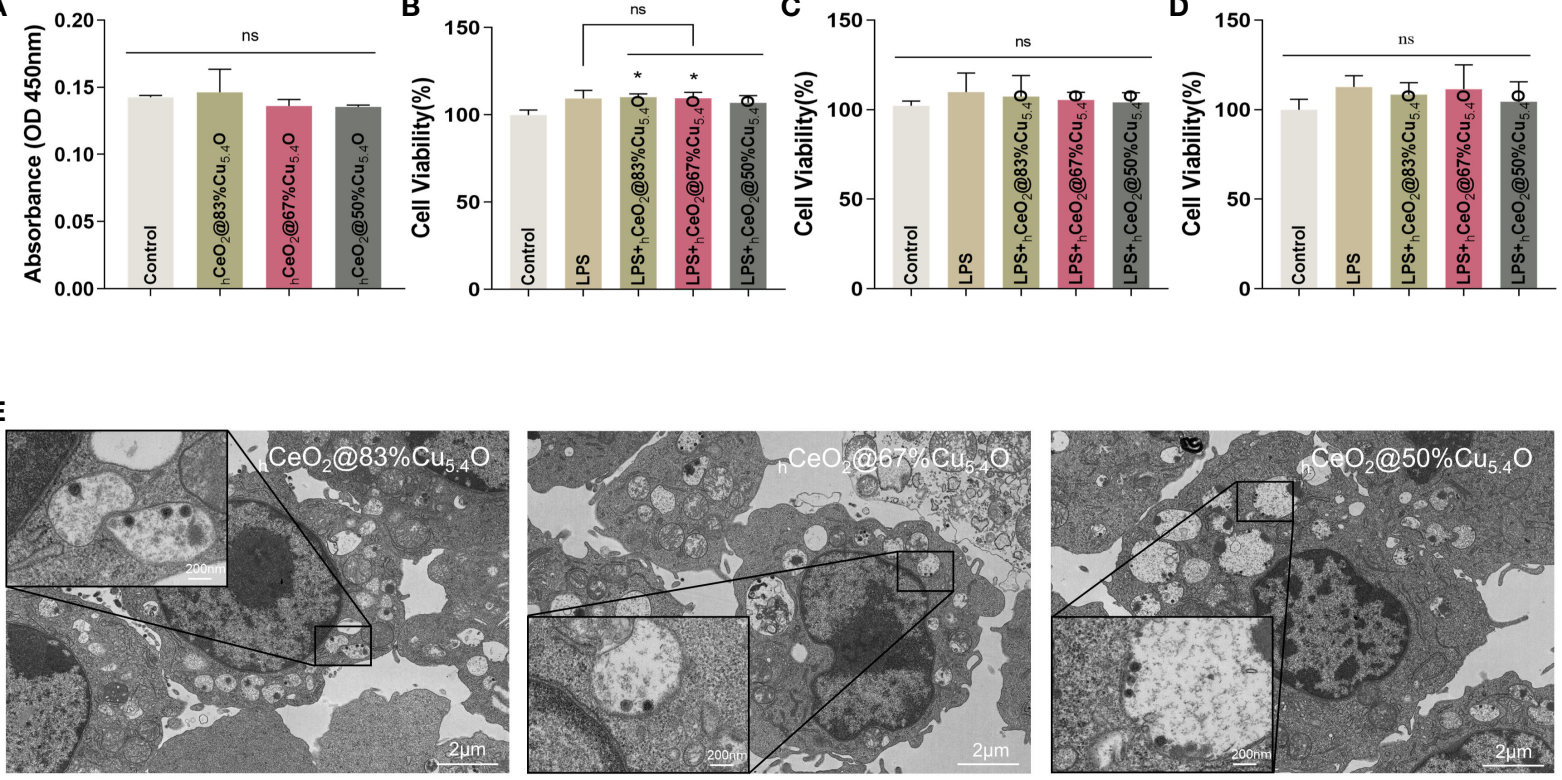
Biocompatibility of _h_CeO_2_@Cu_5.4_O nanoparticles (NPs) treated with lipopolysaccharide (LPS). **(A)** Absorbance values of the culture medium containing _h_CeO_2_@Cu_5.4_O NPs added with CCK8 reagent at 450 nm. **(B–D)** Cell viability of RAW264.7 cells treated with LPS and _h_CeO_2_@Cu_5.4_O NPs for 24, 48, and 72 h. **(E)** Bio-TEM images of _h_CeO_2_@Cu_5.4_O NP cellular uptake. (n=3; ns: no significance; *p<0.05.)

However, it has been reported that nanoparticles may interfere with the results of conventional toxicity measurement through different mechanisms (44–46), such as the absorption and scattering of light at a certain wavelength by nanoparticles or the reaction with substrates to interfere with the absorbance value. In order to exclude the possible interference of nanoparticles on the assay, firstly, we removed the medium containing nanoparticles and cleaned it with PBS before the assay. Secondly, considering the interference of nanoparticles entering the cells, we tested the effect of nanoparticles on the absorbance value of the CCK8 reagent (**Figure 5A**). The results showed that there was no difference in absorbance compared with the CCK8 reagent without nanoparticles.

In the subsequent experiments, LPS and _h_CeO_2_@Cu_5.4_O NPs were used to treat the RAW 264.7 cells, so we also conducted a toxicity test for LPS and _h_CeO_2_@Cu_5.4_O NP co-treatment. The results are shown in **Figures 5B–D**. The cell viability was increased after 1-ug/mL LPS treatment, which was consistent with the previous literature (47), but there was no significant statistical difference. There was no significant change in cell viability after adding 10 mg/L nanoparticles compared with the LPS group. The toxicity of nanoparticles depends on many factors, including their chemical composition, size, and surface properties. It has been reported that the toxicity of metal nanoparticles is mainly derived from the metal ions released from them, and exceeding a certain concentration of metal ions may increase the production of intracellular ROS and the occurrence of cytotoxicity (48, 49). However, in this study, the nanoparticle concentration of 10 mg/L was not toxic to RAW 264.7 at 24, 48, or 72 h.

### 3.4 Uptake of _h_CeO_2_@Cu_5.4_O NPs in RAW264.7 macrophages

Immune cells are the first barrier for nanoparticles to penetrate into cells (50). It is necessary to study the uptake of nanoparticles by macrophages, which is essential for nanoparticles to exert their effects and potentially induce a toxic response. Studies have reported the presence of nanoparticles in the endosome after a 3-h incubation period with cells, forming relatively large and dense aggregates (50). Furthermore, nanoparticles were detectable in the cytoplasm after 24 h (51). Some research suggested that nanoparticles continue to degrade within the cells over time (52).

In our study, we took TEM images of cells treated with nanoparticles for 24 h. As shown in **Figure 5E**, the nucleus and the cytoplasm were clearly visible in the image, and the nanoparticles can be observed in the cytoplasm. The cell membrane folds and fuses to internalize the nanoparticles, and vesicles that internalize nanoparticles, as well as vesicles that internalize particle aggregates, can also be observed in the cytoplasm. This could also indicate that, at the concentration of 10 mg/L, _h_CeO_2_@Cu_5.4_O NPs can enter the cells and provide evidence for the intracellular anti-inflammatory effect. However, some studies have shown that with the increase of nanoparticle concentration, the number and volume of intracellular vesicles increase, which may hinder the function of other organelles and eventually lead to cell death (53). In this study, combined with the screening of the safe concentration of _h_CeO_2_@Cu_5.4_O NPs in the previous 24-, 48-, and 72-h CCK8 experiment, the follow-up anti-inflammatory study could be carried out on the premise that the concentration of 10 mg/L was relatively able to ensure the cell activity.

### 3.5 _h_CeO_2_@Cu_5.4_O NPs reduce NRLP3 inflammasome activation and inflammatory factor expression by reducing CTSB release

#### 3.5.1 Reduced protein expression of CTSB in RAW264.7

The inflammatory cascade initiated by LPS via the toll-like receptor 4/CD14 receptor complex (TLR4) plays a crucial role in LPS-stimulated inflammatory responses. TLR4 recognizes and binds LPS, recruiting and activating the downstream molecule NF-κB, which enters the nucleus and induces the transcription of NLPR3, IL-18, and IL-1β, leading to the release of pro-inflammatory mediators as well as inflammation generation and development (1, 54). Inflammation is a defensive response that protects the host from harmful stimuli of endogenous and exogenous origin. On the other hand, infiltration of inflammatory cells and accumulation of inflammatory factors disrupt the structure and function of normal tissues and promote the development of a variety of inflammatory disorders (1, 55). The NLRP3 inflammatory vesicle plays an important role in the activation of caspase-1 and the subsequent release of inflammatory factors. In a mouse model of periodontitis, researchers found that the knockdown of NLRP3 prevented IL-1β release and inhibited osteoclast differentiation, implying that inflammasome may be closely related to the pathological process of periodontitis (56). There are also many studies showing that NLRP3 is also capable of inducing different aseptic inflammatory disorders, more so than atherosclerosis, lung inflammation due to mechanical distraction, and drug-induced hepatitis (57–59). CTSB, as a class of proteases, is mainly found in lysosomes. However, under certain specific circumstances, lysosomal membrane permeability is enhanced and CTSB is released, thus participating in many pathological mechanisms, especially playing an important role in the development of inflammatory diseases. CTSB has been proposed to promote NLRP3 activation and inflammatory factor production. It has been shown that the expression of CTSB, NLRP3, IL-18, and IL-1β was upregulated in PA-induced inflammation, while the above-mentioned molecules are not significantly different from control after the addition of CTSB inhibitors (60). Therefore, CTSB may regulate IL-18\IL-1β secretion by regulating the NLRP3 inflammasome. In another study, it was found that CTSB/NLRP3 expression was increased in cerulein-induced pancreatitis, and the addition of CA-074Me not only inhibited CTSB activity but also downregulated NLRP3, ASC, and caspase-1 expression. The levels of IL-18 and IL-1β were also significantly reduced in the CA-074Me addition group compared with the control group (61).

In this study, we investigated the expression and interrelationships of CTSB, NLRP3, ASC, and caspase-1 in mouse macrophages in order to understand the possible molecular mechanism of nanoparticles in the inflammatory pathway. The RNA expression of each component of the CTSB–NLRP3 pathway and inflammatory factors IL-18 and IL-1β was evaluated. Our results suggested that LPS-induced cellular inflammation was associated with the CTSB–NLRP3 pathway and that _h_CeO_2_@Cu_5.4_O NPs can alleviate the inflammatory response by inhibiting this signaling pathway.

**Figure 6:**
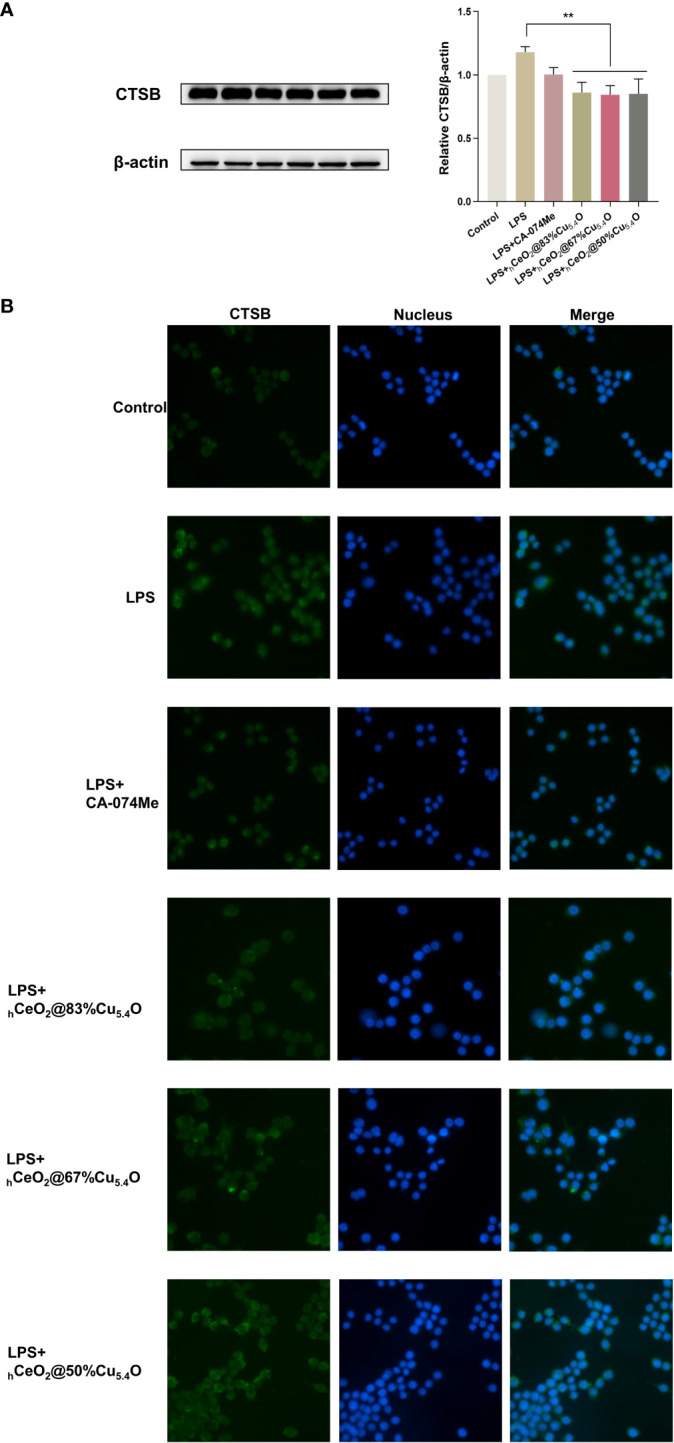
_h_CeO_2_@ Cu_5.4_O nanoparticles (NPs) reduce lipopolysaccharide (LPS)-induced CTSB release in RAW264.7 cells. **(A)** Western blot analysis of CTSB protein expression upon RAW264.7 cells treated with 1 μg/mL LPS and _h_CeO_2_@Cu_5.4_O NPs for 24 h. Data are presented as mean ± SD from three independent experiments. *n* = 3; * represents significant differences. **P< 0.005. **(B)** CTSB immunofluorescent staining.

We used the group of LPS-stimulated cells with inflammatory response as a positive control group. **Figure 6A** shows the Western blot bands and quantitative analysis plots, respectively. We found that LPS stimulation significantly increased the CTSB protein levels, which were significantly reduced after nanomaterial treatment, and the most significant protein reduction was observed when the molar ratio Cu/(Ce+Cu) was 83% (_h_CeO_2_@83%Cu_5.4_O). The localization of CTSB in mouse macrophages was also examined (**Figure 6B**). DAPI was used to show the nucleus marked as blue fluorescence, while CTSB is marked as green fluorescence. The fluorescence images showed an increase in CTSB after LPS stimulation and a decrease in CTSB after nanoparticle treatment. Therefore, we hypothesized that nanoparticles improved the inflammatory response induced by LPS stimulation by reducing the release of CTSB and that _h_CeO_2_@83%Cu_5.4_O inhibited the release of CTSB best.

#### 3.5.2 Inhibiting the CTSB–NLRP3 signaling pathway in LPS-stimulated inflammation model

The specific mechanism of NLRP3 inflammasome activation is still unclear, and the widely accepted model is the double signal model. First, the above-mentioned LPS or other microbial molecules stimulate as the first signal, upregulating the expression of NLRP3 and IL-1β through the transcription factor NF-κB. Signal two can be provided by many stimuli, including ATP, ROS, oxidized mtDNA, K^+^ efflux, lysosomal rupture, CTSB, etc. Among them, CTSB is a lysosomal enzyme that is widely expressed in mammalian cells and is a marker of lysosome-specific damage (62). When the integrity of the lysosomal membrane is disrupted, lysosomal enzymes such as CTSB leak into the cytoplasm, leading to a series of cellular homeostasis imbalances, pathological processes, and cell apoptosis. Studies have found that in microglial cells, CTSB can chronically activate the NF-κB signaling pathway by degrading IκBα (63); and other studies have found that chemical inhibitors of tissue protease B, such as CA-074Me, can inhibit NLRP3 activation, leading to the conclusion that CTSB can affect NLRP3 in different ways, including inhibiting the activation of NF-κB, reducing the expression of NLRP3 genes, and inhibiting the activation of NLRP3 (64, 65).

**Figure 7:**
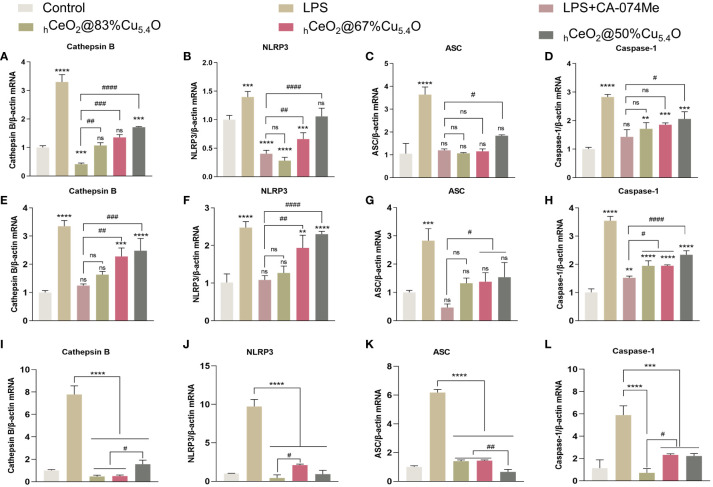
Inhibiting the CTSB–NLRP3 signaling pathway in a lipopolysaccharide (LPS)-stimulated inflammation model. **(A–D)** mRNA expression of IL-18, IL-1b, CTSB, NLRP3, ASC, and caspase-1 in RAW264.7 cells treated with LPS and _h_CeO_2_@Cu_5.4_O nanoparticles (NPs) for 24 h. **(E–H)** mRNA expression of IL-18, IL-1β, CTSB, NLRP3, ASC, and caspase-1 in RAW264.7 cells treated with LPS and _h_CeO_2_@Cu_5.4_O NPs for 12 h. **(I–L)** mRNA expression of CTSB, NLRP3, ASC, and caspase-1 in RAW264.7 cells treated only with _h_CeO_2_@Cu_5.4_O NPs for 24 h.( n = 3; ns: no significance; * and # represent significant differences. **P < 0.005, ***P < 0.0005, ****P < 0.0001, ^#^ *P* < 0.05, ^##^ *P* < 0.005, ^###^ *P* < 0.0005, and ^####^ *P* < 0.0001).

As shown in **Figures 7A–D**, NLRP3, ASC and caspase-1 expression increased after LPS treatment and decreased after the addition of CA-074Me, indicating that the reduction of CTSB reduced the expression of NLRP3 inflammasome. The addition of nanomaterials also reduced the NLRP3 inflammasome components, indicating that nanomaterials reduce the expression of NLRP3 inflammasome by inhibiting CTSB and reduce the release of inflammatory factors, thereby reducing the inflammatory response. Moreover, when the Ce/Cu ratio was 0.2 (_h_CeO_2_@83%Cu_5.4_O), the effect on inflammation was the best, and with the decrease of Cu doping, the inhibitory effect on inflammation was weakened. In addition, **Figures 7E–H** show the gene expression of cells treated with LPS and nanoparticles for 12 h. The results show that the anti-inflammatory effect of cells treated with LPS and nanoparticles for 12 h is about the same as that of cells treated for 24 h, but the anti-inflammatory effect is not as good as that treated with LPS for 24 h. As shown in **Figures 7I–L**, to further understand the effect of nanoparticles on the CTSB–NLRP3 signaling pathway, we treated cells with nanoparticles only for 24 h and that _h_CeO_2_@Cu_5.4_O showed an obvious inhibitory effect to NLRP3 inflammasome-related factor gene expression. From the above-mentioned experiments, _h_CeO_2_@83%Cu_5.4_O NPs had the best effect.

We believe the possible reasons for this phenomenon were as follows: (1) Cu in Cu_5.4_O NPs could reduce Ce^4+^ to Ce^3+^. The more Cu_5.4_O NPs doped, the more Ce^3+^ is reduced, which exerts better SOD activity and reduces inflammation. (2) The anti-inflammatory effect of Cu_5.4_O NPs: Their ultra-small size and more active site exposure give them ultra-high antioxidant capacity. The particle size of Cu_5.4_O NPs was mostly 3–5 nm, which belongs to ultra-small nanoparticles. It has been reported that it can enter the mitochondrial permeability transition pore, thus maintaining the normal function of the mitochondria and alleviating oxidative stress. It can also enter the cells to play a role through phagocytosis (16). Thus, the increase in Cu_5.4_O NPs increases its antioxidant and anti-inflammatory effects.

**Figure 8:**
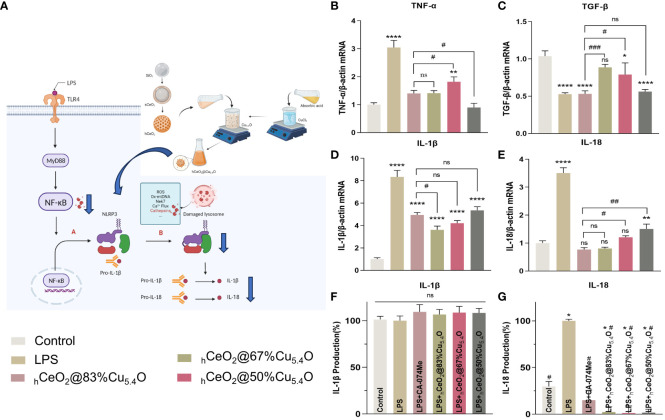
Secretion of IL-18 and IL-1β pro-inflammatory cytokines. **(A)** Schematic representation of _h_CeO_2_@Cu_5.4_O nanoparticles (NPs) on the CTSB–NLRP3 signaling pathway. **(B, C)** mRNA expression of TNF-a and TGF-β in RAW264.7 cells treated with _h_CeO_2_@Cu_5.4_O NPs for 24 h. **(D, E)** mRNA expression of IL-18 and IL-1β in RAW264.7 cells treated with _h_CeO_2_@Cu_5.4_O NPs for 24 h. **(F, G)** Effects of _h_CeO_2_@Cu_5.4_O NPs on the secretion of IL-18 and IL-1β in LPS-induced RAW264.7 cell supernatant. (n = 3; ns: no significance; * and # represent significant differences. *P < 0.05, **P < 0.005, ****P < 0.0001, ^#^ *P* < 0.05, ^##^ *P* < 0.005, ^###^ *P* < 0.0005).

In general, LPS can stimulate macrophages to M1 polarization, recruit inflammatory cells, secrete inflammatory factors, and facilitate the clearance of pathogens. M2 macrophages play an important role in resolving inflammation and tissue repair (66). We also tested the effect of _h_CeO_2_@Cu_5.4_O NPs on the gene expression of TNF-α and TGF-β, and as shown in **Figures 8B, C**, _h_CeO_2_@Cu_5.4_O NPs were able to reduce the expression of TNF-α and increase the expression of TGF-β, further suggesting that _h_CeO_2_@Cu_5.4_O NPs may alleviate inflammation by promoting macrophage polarization to M2 type.

#### 3.5.3 The secretion of IL-18 and IL-1β pro-inflammatory cytokines

After activation of NLRP3 inflammasome, pro-IL-18 and pro-IL-1β could be cleaved into mature IL-18 and IL-1β (**Figures 8A**) (67). As shown in **Figures 8D, E**, the mRNA expression levels of the inflammatory factors IL-18 and IL-1β were increased in the LPS-treated group, while their expression was decreased by the addition of CA-074Me. These results indicated that CTSB is involved in LPS-induced inflammatory response. The expression levels of all CTSB, IL-18, and IL-1β decreased after adding _h_CeO_2_@Cu_5.4_O, indicating that _h_CeO_2_@Cu_5.4_O may alleviate inflammatory response by inhibiting CTSB.

To evaluate the effect of nanoparticles on the secretion of IL-18 and IL-1β in the culture supernatant of macrophages, the levels of IL-18 and IL-1β in the culture supernatant of RAW 264.7 macrophages were analyzed. As shown in **Figures 8F, G**, IL-18 production was significantly increased in the LPS group compared with the control group. Compared with the LPS group, the groups treated with different _h_CeO_2_@Cu_5.4_O showed a significant reduction in IL-18 level. However, there was no significant difference in the IL-1β levels among the groups. It has been documented that IL-1β production is dependent on ASC that is not expressed in RAW264.7 (68, 69). Studies have shown that some pro-IL-1β is released into the culture medium after RAW264.7 is stimulated, and most pro-IL-1β is retained in the cells. When co-stimulated with other stimuli, pro-IL-1β and IL-1β are released from the cells together (69). However, other studies have shown that the expression of IL-1β in the supernatant of RAW264.7 cells treated with LPS increased (70, 71). In the future, we need to combine LPS- and NLRP3-specific agonists to further explore the inhibitory effect on IL-1β.

## 4 Conclusion

In this study, a novel _h_CeO_2_@Cu_5.4_O NPs was prepared by doping different amounts of Cu_5.4_O NPs into hCeO_2_ NPs. _h_CeO_2_@Cu_5.4_O NPs has good biocompatibility and excellent ROS scavenging ability. _h_CeO_2_ @ Cu_5.4_O NPs was demonstrated to attenuate the inflammatory response by scavenging ROS, reducing the release of CTSB, and inhibiting the activation of the NLPR3 inflammasome. _h_CeO_2_@Cu_5.4_O NPs could alleviate the inflammatory responses by regulating the CTSB–NLRP3 signaling pathway, where the _h_CeO_2_@83%Cu_5.4_O NPs had the strongest antioxidant and anti-inflammatory effects. This study provides a new idea for nanoparticles to attenuate LPS-induced inflammatory response.

## Data availability statement

The original contributions presented in the study are included in the article/supplementary material. Further inquiries can be directed to the corresponding authors.

## Ethics statement

Ethical approval was not required for the studies on animals in accordance with the local legislation and institutional requirements because only commercially available established cell lines were used.

## Author contributions

YL: Writing – original draft. XX: Writing – review & editing. ZN: Writing – review & editing. KW: Writing – review & editing. JL: Writing – review & editing. XL: Writing – review & editing.

## References

[1] Afsar UA. An overview of inflammation:mechanism and consequences. Front Biol. (2011) 6:274–81. doi: 10.1007/s11515-011-1123-9

[2] Nathan C Ding AH. Nonresolving inflammation. Cell. (2010) 140:871–82. doi: 10.1016/j.cell.2010.02.029 20303877

[3] Weber C Hristov M. Atherogenesis and inflammation. Hamostaseologie. (2015) 35:99. doi: 10.1055/s-0037-1619816 25943077

[4] Singh N Baby D Rajguru JP Patil PB Thakkannavar SS Pujari VB. Inflammation and cancer. Ann Afr Med. (2019) 18:121–6. doi: 10.4103/aam.aam_56_18 PMC6704802 31417011

[5] Xie W Du L. Diabetes is an inflammatory disease: evidence from traditional Chinese medicines. Diabetes Obes Metab. (2011) 13(4):289–301. doi: 10.1111/j.1463-1326.2010.01336.x 21205111

[6] Algarni A Fayomi A Al Garalleh H Afandi A Brindhadevi K Pugazhendhi A. Nanofabrication synthesis and its role in antibacterial, anti-inflammatory, and anticoagulant activities of AgNPs synthesized by Mangifera indica bark extract. Environ Res. (2023) 231:115983. doi: 10.1016/j.envres.2023.115983 37137456

[7] Uchiyama MK Deda DK Rodrigues SFD Drewes CC Bolonheis SM Kiyohara PK. *In vivo* and *in vitro* toxicity and anti-inflammatory properties of gold nanoparticle bioconjugates to the vascular system. Toxicological Sci. (2014) 142:497–507. doi: 10.1093/toxsci/kfu202 25260831

[8] Agarwal H Nakara A Shanmugam VK. Anti-inflammatory mechanism of various metal and metal oxide nanoparticles synthesized using plant extracts: A review. Biomedicine Pharmacotherapy. (2019) 109:2561–72. doi: 10.1016/j.biopha.2018.11.116 30551516

[9] Tripathi P Tripathi P Kashyap L Singh V. The role of nitric oxide in inflammatory reactions. FEMS Immunol Med Microbiol. (2007) 51:443–52. doi: 10.1111/j.1574-695X.2007.00329.x 17903207

[10] Agrawal G Aswath S Laha A Ramakrishna S. Electrospun nanofiber-based drug carrier to manage inflammation. Adv Wound Care (New Rochelle). (2023) 12:529–43. doi: 10.1089/wound.2022.0043 36680757

[11] Luo W Bai L Zhang J Li Z Liu Y Tang X. Polysaccharides-based nanocarriers enhance the anti-inflammatory effect of curcumin. Carbohydr polymers. (2023) 311:120718. doi: 10.1016/j.carbpol.2023.120718 37028867

[12] Qi M Ren X Li W Sun Y Sun X Li C. NIR responsive nitric oxide nanogenerator for enhanced biofilm eradication and inflammation immunotherapy against periodontal diseases. Nano Today. (2022) 43:101447. doi: 10.1016/j.nantod.2022.101447

[13] Sadidi H Hooshmand S Ahmadabadi A Javad Hoseini S Baino F Vatanpour M. Cerium oxide nanoparticles (Nanoceria): hopes in soft tissue engineering. Molecules. (2020) 25(19):4559. doi: 10.3390/molecules25194559 33036163 PMC7583868

[14] Arya A Sethy NK Singh SK Das M Bhargava K. Cerium oxide nanoparticles protect rodent lungs from hypobaric hypoxia-induced oxidative stress and inflammation. Int J Nanomedicine. (2013) 8:4507–19. doi: 10.2147/IJN PMC3839803 24294000

[15] Yu Y Zhao S Gu D Zhu B Liu H Wu W. Cerium oxide nanozyme attenuates periodontal bone destruction by inhibiting the ROS-NFκB pathway. Nanoscale. (2022) 14:2628–37. doi: 10.1039/D1NR06043K 35088792

[16] Liu TF Xiao BW Xiang F Tan JL Chen Z Zhang XR. Ultrasmall copper-based nanoparticles for reactive oxygen species scavenging and alleviation of inflammation related diseases. Nat Commun. (2020) 11(1):2788. doi: 10.1038/s41467-020-16544-7 32493916 PMC7270130

[17] Xiong J Wang Y Xue Q Wu X. Synthesis of highly stable dispersions of nanosized copper particles using L-ascorbic acid. Green Chem. (2011) 13:900–4. doi: 10.1039/c0gc00772b

[18] Ingle AP Paralikar P Shende S Gupta I Biswas JK da Silva Martins LH. Copper in medicine: Perspectives and toxicity. In: Rai M Ingle AP Medici S, eds. Biomedical Applications of Metals. Cham: Springer International Publishing (2018). pp. 95–112. doi: 10.1007/978-3-319-74814-6_4

[19] Bergsbaken T Fink SL Cookson BT. Pyroptosis: host cell death and inflammation. Nat Rev Microbiol. (2009) 7:99–109. doi: 10.1038/nrmicro2070 19148178 PMC2910423

[20] Jiang Y Wang M Huang K Zhang Z Shao N Zhang Y. Oxidized low-density lipoprotein induces secretion of interleukin-1β by macrophages via reactive oxygen species-dependent NLRP3 inflammasome activation. Biochem Biophys Res Commun. (2012) 425:121–6. doi: 10.1016/j.bbrc.2012.07.011 22796220

[21] Man SM Kanneganti T-D. Regulation of lysosomal dynamics and autophagy by CTSB/cathepsin B. Autophagy. (2016) 12:2504–5. doi: 10.1080/15548627.2016.1239679 PMC5173259 27786577

[22] Wang Y Jia L Shen J Wang Y Fu Z S-a Su. Cathepsin B aggravates coxsackievirus B3-induced myocarditis through activating the inflammasome and promoting pyroptosis. Plos Pathogens. (2018) 14(1):e1006872. doi: 10.1371/journal.ppat.1006872 29360865 PMC5809100

[23] Menzel K Hausmann M Obermeier F Schreiter K Dunger N Bataille F. Cathepsins B, L and D in inflammatory bowel disease macrophages and potential therapeutic effects of cathepsin inhibition *in vivo* . Clin Exp Immunol. (2006) 146:169–80. doi: 10.1111/j.1365-2249.2006.03188.x PMC1809720 16968411

[24] Ma X Cheng Y Jian H Feng Y Chang Y Zheng R. Hollow, rough, and nitric oxide-releasing cerium oxide nanoparticles for promoting multiple stages of wound healing. Advanced healthcare materials. (2019) 8:e1900256. doi: 10.1002/adhm.201900256 31290270

[25] Li F Li J Song X Sun T Mi L Liu J. Alginate/Gelatin hydrogel scaffold containing nCeO2 as a potential osteogenic nanomaterial for bone tissue engineering. Int J Nanomedicine. (2022) 17:6561–78. doi: 10.2147/IJN.S388942 PMC9791564 36578441

[26] Michael JM Liu Z. Crosstalk of reactive oxygen species and NF-KB signaling. Cell Res. (2011) 21:103–15. doi: 10.1038/cr.2010.178 PMC3193400 21187859

[27] Codolo G Plotegher N Pozzobon T Brucale M Tessari I Bubacco L. Triggering of inflammasome by aggregated alpha-synuclein, an inflammatory response in synucleinopathies. PloS One. (2013) 8(1):e55375. doi: 10.1371/journal.pone.0055375 23383169 PMC3561263

[28] Liu X Wu J Liu Q Lin A Li S Zhang Y. Synthesis-temperature-regulated multi-enzyme-mimicking activities of ceria nanozymes. J Materials Chem B. (2021) 9:7238–45. doi: 10.1039/D1TB00964H 34095923

[29] Dong HJ Zhang C Fan YY Zhang W Gu N Zhang Y. Nanozyme and their ROS regulation effect in cells. Prog Biochem Biophysics. (2018) 45:105–17. doi: 10.16476/j.pibb.2017.0460

[30] Yasui K Baba A. Therapeutic potential of superoxide dismutase (SOD) for resolution of inflammation. Inflammation Res. (2006) 55:359–63. doi: 10.1007/s00011-006-5195-y 17122956

[31] Celardo I Pedersen JZ Traversa E Ghibelli L. Pharmacological potential of cerium oxide nanoparticles. Nanoscale. (2011) 3:1411–20. doi: 10.1039/c0nr00875c 21369578

[32] Zeng F Shi YH Wu CN Liang JM Zhong QX Briley K. A drug-free nanozyme for mitigating oxidative stress and inflammatory bowel disease. J Nanobiotechnology. (2022) 20(1):107. doi: 10.1186/s12951-022-01319-7 35246140 PMC8896226

[33] Li MY Liu J Shi L Zhou C Zou MZ Fu D. Gold nanoparticles-embedded ceria with enhanced antioxidant activities for treating inflammatory bowel disease. Bioact Mater. (2023) 25:95–106. doi: 10.1016/j.bioactmat.2023.01.015 36789001 PMC9900456

[34] Kim J Kim HY Song SY Go SH Sohn HS Baik S. Synergistic oxygen generation and reactive oxygen species scavenging by manganese Ferrite/Ceria co-decorated nanoparticles for rheumatoid arthritis treatment. ACS Nano. (2019) 13:3206–17. doi: 10.1021/acsnano.8b08785 30830763

[35] Shlapa Y Solopan S Sarnatskaya V Siposova K Garcarova I Veltruska K. Cerium dioxide nanoparticles synthesized via precipitation at constant pH: Synthesis, physical-chemical and antioxidant properties. Colloids Surfaces B: Biointerfaces. (2022) 220:112960. doi: 10.1016/j.colsurfb.2022.112960 36308885

[36] Gupta A Das S Neal CJ Seal S. Controlling the surface chemistry of cerium oxide nanoparticles for biological applications. J Materials Chem B. (2016) 4:3195–202. doi: 10.1039/C6TB00396F 32263255

[37] Qiao XJ Arsalan M Ma X Wang YH Yang SY Wang Y. A hybrid of ultrathin metal-organic framework sheet and ultrasmall copper nanoparticles for detection of hydrogen peroxide with enhanced activity. Anal Bioanal Chem. (2021) 413:839–51. doi: 10.1007/s00216-020-03038-0 33219832

[38] Peng Y He DF Ge X Lu YF Chai YH Zhang YX. Construction of heparin-based hydrogel incorporated with Cu5.4O ultrasmall nanozymes for wound healing and inflammation inhibition. Bioact Mater. (2021) 6:3109–24. doi: 10.1016/j.bioactmat.2021.02.006 PMC7960791 33778192

[39] Gough DR Cotter TG. Hydrogen peroxide: a Jekyll and Hyde signalling molecule. Cell Death Dis. (2011) 2(10):e213. doi: 10.1038/cddis.2011.96 21975295 PMC3219092

[40] Bryan N Ahswin H Smart N Bayon Y Wohlert S Hunt JA. Reactive oxygen species (ROS) - a family of fate deciding molecules pivotal in constructive inflammation and wound healing. Eur Cells Materials. (2012) 24:249–65. doi: 10.22203/eCM 23007910

[41] Rimessi A Previati M Nigro F Wieckowskic MR Pinton P. Mitochondrial reactive oxygen species and inflammation: Molecular mechanisms, diseases and promising therapies. Int J Biochem Cell Biol. (2016) 81:281–93. doi: 10.1016/j.biocel.2016.06.015 27373679

[42] Kavcic N Pegan K Turk B. Lysosomes in programmed cell death pathways: from initiators to amplifiers. Biol Chem. (2017) 398:289–301. doi: 10.1515/hsz-2016-0252 28002019

[43] Valodkar M Rathore PS Jadeja RN Thounaojam M Devkar RV Thakore S. Cytotoxicity evaluation and antimicrobial studies of starch capped water soluble copper nanoparticles. J Hazard Mater. (2012) 201:244–9. doi: 10.1016/j.jhazmat.2011.11.077 22178277

[44] Andraos C Yu IJ Gulumian M. Interference: A much-neglected aspect in high-throughput screening of nanoparticles. Int J Toxicol. (2020) 39:397–421. doi: 10.1177/1091581820938335 32672081

[45] Guadagnini R Kenzaoui BH Walker L Pojana G Magdolenova Z Bilanicova D. Toxicity screenings of nanomaterials: challenges due to interference with assay processes and components of classic *in vitro* tests. Nanotoxicology. (2015) 9:13–24. doi: 10.3109/17435390.2013.829590 23889211

[46] Drasler B Sayre P Steinhaeuser KG Petri-Fink A Rothen-Rutishauser B. *In vitro* approaches to assess the hazard of nanomaterials (vol 8, pg 99, 2017). Nanoimpact. (2018) 9:51–1. doi: 10.1016/j.impact.2017.10.002

[47] Yin Q Jiang D Li L Yang Y Wu P Luo Y. LPS promotes vascular smooth muscle cells proliferation through the TLR4/Rac1/Akt signalling pathway. Cell Physiol Biochem. (2017) 44:2189–200. doi: 10.1159/000486024 29298445

[48] Horie M Tabei Y. Role of oxidative stress in nanoparticles toxicity. Free Radical Res. (2021) 55:331–42. doi: 10.1080/10715762.2020.1859108 33336617

[49] Chang Y-N Zhang M Xia L Zhang J Xing G. The toxic effects and mechanisms of CuO and ZnO nanoparticles. Materials. (2012) 5:2850–71. doi: 10.3390/ma5122850

[50] Mi X-J Xu XY Choi HS Kim H Cho IH Yi T-H. The immune-enhancing properties of hwanglyeonhaedok-tang-mediated biosynthesized gold nanoparticles in macrophages and splenocytes. Int J Nanomedicine. (2022) 17:477–94. doi: 10.2147/IJN.S338334 PMC8812323 35125869

[51] Lee JK Sayers BC Chun K-S Lao H-C Shipley-Phillips JK Bonner JC. Multi-walled carbon nanotubes induce COX-2 and iNOS expression via MAP Kinase-dependent and -independent mechanisms in mouse RAW264.7 macrophages. Particle Fibre Toxicol. (2012) 9(1):14. doi: 10.1186/1743-8977-9-14 PMC3485091 22571318

[52] Zhang L Xiao S Kang X Sun T Zhou C Xu Z. Metabolic conversion and removal of manganese ferrite nanoparticles in RAW264.7 cells and induced alteration of metal transporter gene expression. Int J Nanomedicine. (2021) 16:1709–24. doi: 10.2147/IJN.S289707 PMC7936572 33688187

[53] Hashimoto M Toshima H Yonezawa T Kawai K Narushima T Kaga M. Responses of RAW264.7 macrophages to water-dispersible gold and silver nanoparticles stabilized by metal-carbon σ-bonds. J BioMed Mater Res A. (2014) 102:1838–49. doi: 10.1002/jbm.a.34854 23784947

[54] Liu C Yao Q Hu TT Cai ZL Xie QW Zhao JH. Cathepsin B deteriorates diabetic cardiomyopathy induced by streptozotocin via promoting NLRP3-mediated pyroptosis. Mol Therapy-Nucleic Acids. (2022) 30:198–207. doi: 10.1016/j.omtn.2022.09.019 PMC9554743 36250207

[55] Rubartelli A Lotze MT Latz E Manfredi AJ. Mechanisms of sterile inflammation. Front Immunol. (2013) 4:398. doi: 10.3389/fimmu.2013.00398 24319446 PMC3837241

[56] Chen Y Yang Q Lv C Chen Y Zhao W Li W. NLRP3 regulates alveolar bone loss in ligature-induced periodontitis by promoting osteoclastic differentiation. Cell Proliferation. (2021) 54(2):e12973. doi: 10.1111/cpr.12973 33382502 PMC7849172

[57] Chen GY Nuñez G. Sterile inflammation: sensing and reacting to damage. Nat Rev Immunol. (2010) 10(12):826–37. doi: 10.1038/nri2873 PMC3114424 21088683

[58] Duewell P Kono H Rayner KJ Sirois CM Vladimer G Bauernfeind FG. NLRP3 inflammasomes are required for atherogenesis and activated by cholesterol crystals. Nature. (2010) 464:1357–U1357. doi: 10.1038/nature08938 20428172 PMC2946640

[59] Imaeda AB Watanabe A Sohail MA Mahmood S Mohamadnejad M Sutterwala FS. Acetaminophen-induced hepatotoxicity in mice is dependent on Tlr9 and the Nalp3 inflammasome. J Clin Invest. (2009) 119:305–14. doi: 10.1172/JCI35958 PMC2631294 19164858

[60] Tang Y Cao G Min X Wang T Sun S Du X. Cathepsin B inhibition ameliorates the non-alcoholic steatohepatitis through suppressing caspase-1 activation. J Physiol Biochem. (2018) 74:503–10. doi: 10.1007/s13105-018-0644-y 30019185

[61] Wang J Wang L Zhang X Xu Y Chen L Zhang W. Cathepsin B aggravates acute pancreatitis by activating the NLRP3 inflammasome and promoting the caspase-1-induced pyroptosis. Int Immunopharmacol. (2021) 94:107496. doi: 10.1016/j.intimp.2021.107496 33639565

[62] Lunov O Uzhytchak M Smolková B Lunova M Jirsa M Dempsey NM. Remote actuation of apoptosis in liver cancer cells via magneto-mechanical modulation of iron oxide nanoparticles. Cancers (Basel). (2019) 11(12):1873. doi: 10.3390/cancers11121873 31779223 PMC6966689

[63] Ni JJ Wu Z Peterts C Yamamoto K Qing H Nakanishi H. The critical role of proteolytic relay through cathepsins B and E in the phenotypic change of microglia/macrophage. J Neurosci. (2015) 35:12488–501. doi: 10.1523/JNEUROSCI.1599-15.2015 PMC6605404 26354916

[64] He Y Hara H Núñez G. Mechanism and regulation of NLRP3 inflammasome activation. Trends Biochem Sci. (2016) 41:1012–21. doi: 10.1016/j.tibs.2016.09.002 PMC5123939 27669650

[65] Kelley N Jeltema D Duan Y He Y. The NLRP3 inflammasome: an overview of mechanisms of activation and regulation. Int JMol Sci. (2019) 20(13):3328. doi: 10.3390/ijms20133328 31284572 PMC6651423

[66] Jeganathan S Fiorino C Naik U Sun HS Harrison RE. Modulation of osteoclastogenesis with macrophage M1-and M2-inducing stimuli. PloS One. (2014) 9(8):e104498. doi: 10.1371/journal.pone.0104498 25101660 PMC4125219

[67] Swanson KV Deng M Ting JPY. The NLRP3 inflammasome: molecular activation and regulation to therapeutics. Nat Rev Immunol. (2019) 19:477–89. doi: 10.1038/s41577-019-0165-0 PMC7807242 31036962

[68] Pelegrin P Barroso-Gutierrez C Surprenant A. P2X7 Receptor differentially couples to distinct release pathways for IL-1β in mouse macrophage. J Immunol. (2008) 180:7147–57. doi: 10.4049/jimmunol.180.11.7147 18490713

[69] Hirano S Zhou Q Furuyama A Kanno S. Differential regulation of IL-1β and IL-6 release in murine macrophages. Inflammation. (2017) 40:1933–43. doi: 10.1007/s10753-017-0634-1 28766178

[70] Xiang P Chen T Mou Y Wu H Xie P Lu G. NZ suppresses TLR4/NF-κB signalings and NLRP3 inflammasome activation in LPS-induced RAW264.7 macrophages. Inflammation Res. (2015) 64:799–808. doi: 10.1007/s00011-015-0863-4 26298161

[71] Xie Q Shen W-W Zhong J Huang C Zhang L Li J. Lipopolysaccharide/adenosine triphosphate induces IL-1β and IL-18 secretion through the NLRP3 inflammasome in RAW264.7 murine macrophage cells. Int J Mol Med. (2014) 34:341–9. doi: 10.3892/ijmm.2014.1755 24789624

